# Tuberculosis-Associated Hemophagocytic Lymphohistiocytosis with Bone Marrow Involvement: Case Report and Literature Review

**DOI:** 10.3390/jcm15218240

**Published:** 2026-10-23

**Authors:** Yixian You, He Li, Xiong Pei, Dongbo Wu, Xiaoju Lv, Yu Wu

**Affiliations:** 1 Center of Infectious Diseases, West China Hospital of Sichuan University, Chengdu 610041, China; yyx981104@163.com (Y.Y.); peixion@163.com (X.P.); dongbohuaxi@scu.edu.cn (D.W.); 2 Department of Hematology and Hematology Research Institute, West China Hospital of Sichuan University, Chengdu 610041, China; lihe@wchscu.cn

**Keywords:** hemophagocytic lymphohistiocytosis, bone marrow tuberculosis, *Mycobacterium tuberculosis*

## Abstract

Tuberculosis is a rare but fatal trigger of secondary hemophagocytic lymphohistiocytosis (HLH). Its diagnosis is challenging because clinical manifestations overlap with HLH, and immunological tests may be falsely negative in HLH-related immune dysfunction. We report a case of a 34-year-old male with fever and pancytopenia. The patient’s diagnostic course was protracted and misleading: all conventional tuberculosis immunological tests were negative, and he initially did not meet HLH-2004 criteria. The diagnosis of TB-HLH was eventually established by a second bone marrow biopsy and metagenomic next-generation sequencing. We also reviewed 66 TB-HLH cases, revealing frequent negative immunological test results and showing that timely anti-tuberculosis therapy was associated with reduced mortality (78.3% vs. 15.0%, *p* < 0.001). This case and our review underscore the importance of early bone marrow biopsy and molecular testing in unexplained HLH, particularly when tuberculosis tests are negative. Given the high mortality and frequent diagnostic delay, clinicians should maintain a high index of suspicion and pursue aggressive diagnostic workup for tuberculosis in HLH patients without a clear trigger. Furthermore, we emphasize the need to raise awareness of TB-HLH and to interpret immunological test results with caution in HLH patients, as negative findings should not be used to exclude tuberculosis.

## 1. Introduction

Hemophagocytic lymphohistiocytosis (HLH) is a life-threatening clinical condition characterized by uncontrolled immune activation and hyperinflammation, resulting in end-organ tissue damage and high mortality rates in untreated patients.

On the basis of etiology, HLH is broadly classified into primary HLH and secondary HLH. Primary HLH is a group of rare inherited disorders related to mutations that impair normal immune cell function, while secondary HLH can be a complication secondary to multiple reasons, such as infectious disease, connective tissue diseases, hematological disease, drugs and malignancy [1]. The clinical importance of HLH has been increasingly recognized in recent years. During the last 20 years, the incidence of HLH has shown a rising trend in different regions, which might be ascribed to increased awareness and diagnosis of this disease [1].

The management of HLH requires a dual approach: anti-inflammatory and supportive therapies to control the life-threatening hyperinflammatory state, and definitive treatment of the underlying trigger, which is the key to improving patient survival and achieving cure. However, this approach presupposes timely identification of the trigger, which is often the most challenging part of the diagnostic process. In many cases, the underlying etiology remains elusive, leading to disease escalation and unfavorable outcomes.

Among triggers of secondary HLH, infection is the most common. Many pathogens can trigger HLH; viruses account for the majority of cases, and Epstein–Barr virus is the most common and notorious trigger. Bacterial infections are less commonly triggers of HLH and generally occur in the setting of severe infection. Among these, TB HLH is particularly elusive and rarely reported. Known as a great mimicker, tuberculosis shares a broad symptom spectrum that overlaps with HLH, and HLH may mask the underlying tuberculous etiology in cases without specific signs of TB, posing a great diagnostic challenge and often leading to poor prognosis. This challenge is compounded by the fact that conventional immunological tests for tuberculosis, such as TST and IGRA, may be falsely negative in the setting of HLH-related immune dysfunction.

In this paper, we report a case of secondary HLH initially mimicking a hematological disease, which ultimately proved to be tuberculosis with bone marrow involvement. By combining this case with a literature review of 66 TB-HLH cases, we aim to (1) illustrate the diagnostic pitfalls of TB-HLH, particularly when immunological tests are negative; (2) emphasize the diagnostic value of bone marrow biopsy and molecular testing; and (3) highlight the prognostic importance of timely anti-tuberculosis therapy.

## 2. Case Presentation

**Figure 1:**
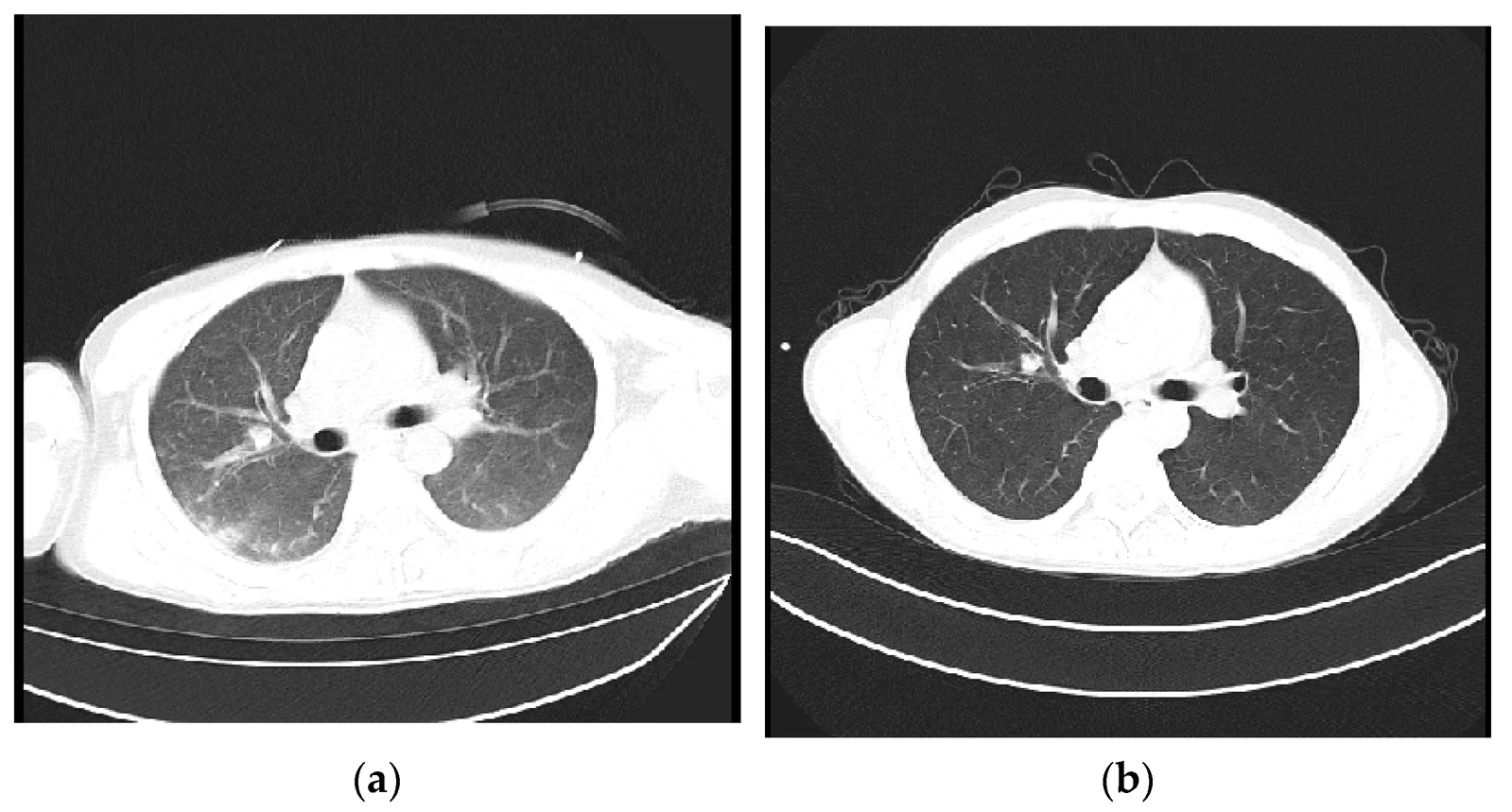
Chest CT image of patient on (**a**) day 1 of admission and (**b**) day 13 of admission, during experimental therapy.

A 34-year-old Tibetan male presented to our hospital with symptoms of intermittent fever, fatigue, skin ecchymosis and epistaxis. Physical examination revealed no splenomegaly, hepatomegaly, or lymphadenopathy. The initial laboratory examination showed severe pancytopenia, with hemoglobin 52 g/L, WBC 0.37 × 10^9^/L and platelet count 2 × 10^9^/L, accompanied by impaired liver function (ALT 147 IU/L, ALT 123 IU/L, LDH 536 IU/L) and elevated procalcitonin. CT scan showed bilateral pulmonary scattered infectious foci and a nodule in the apical segment of the right upper lobe (Figure 1). The patient reported no family history of known hematological disease or tuberculosis. For tuberculosis screening, three consecutive sputum smears for acid-fast bacilli (AFB) were negative. Mycobacterial culture of sputum was also negative. Xpert MTB/RIF (Cepheid, Sunnyvale, CA, USA) and TB-PCR were not performed. Tuberculin skin test (TST), tuberculosis interferon-gamma release assay (TB-IGRA), and tuberculosis antibody test were also negative. Blood culture was negative. HIV screening was negative.

In the emergency department, empiric antibacterial therapy was initiated with piperacillin/tazobactam and meropenem, together with dexamethasone, leukocyte growth factors, blood component transfusions, and hepatoprotective drugs as symptomatic and supportive care. The patient remained in the emergency department for 4 days before being transferred to the Department of Hematology. Considering potential hematological disease, after bone marrow aspiration (on day 0 of hospitalization), the patient was transferred to the Department of Hematology.

The patient’s fever and respiratory symptoms continued, and he subsequently developed hemoptysis. Blood culture yielded *Staphylococcus hominis.* The anti-infective regimen was therefore adjusted to voriconazole in combination with meropenem and vancomycin on day 5.

**Figure 2:**
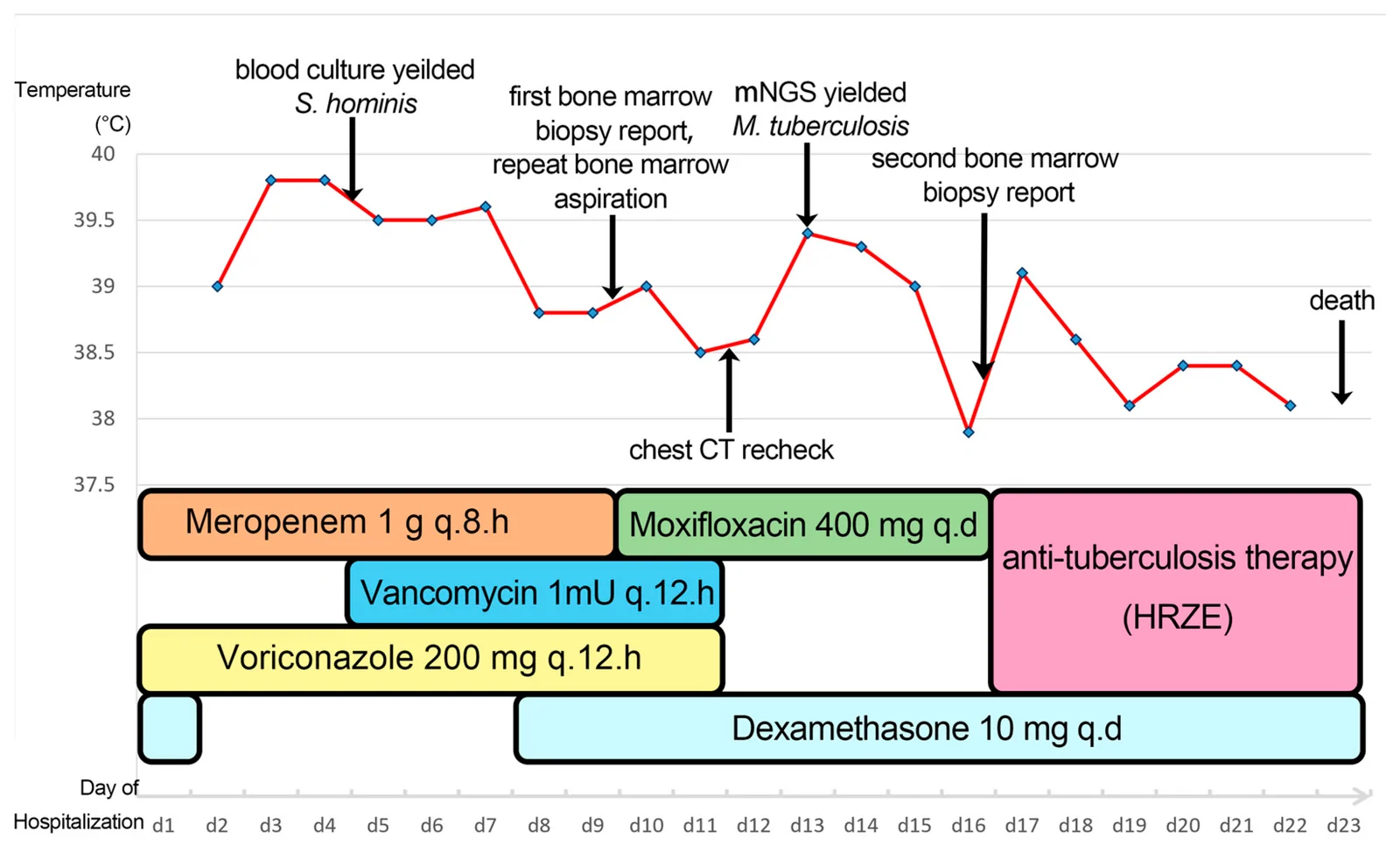
Clinical timeline of the patient, including body temperature peak, major medications, and key events.

With coverage against Gram-positive and Gram-negative bacteria and fungi, his respiratory symptoms improved, and the decreases in procalcitonin and C-reactive protein (CRP) indicated that the infection was controlled, with CRP decreasing from 95.2 mg/L on admission to 37.3 mg/L on day 13 and procalcitonin decreasing from 1.11 ng/mL on admission to 0.21 ng/mL on day 13. However, his fever persisted (Figure 2), with sustained elevations of Soluble interleukin-2 receptor (sIL-2R) and ferritin. sIL-2R increased from 5175 U/mL on day 1 to 7164 U/mL on day 4. Ferritin remained persistently elevated, measuring 4354 ng/mL on day 1, 3727 ng/mL on day 4, and 3993 ng/mL on day 6. By that time, according to the HLH-2004 guidelines (HLH-2024 had not been released by that time), the patient did not meet the diagnostic criteria for HLH, as he met only four of the eight criteria (Table 1). Nevertheless, given the persistent fever, progressive cytopenia, and markedly elevated ferritin and sIL-2R, we maintained a high suspicion of HLH and initiated dexamethasone as empirical immunomodulatory therapy while continuing the diagnostic workup.

Bone marrow biopsy returned on day 9 of hospitalization and indicated no signs of leukemia, lymphoma, or myelodysplastic syndromes. The bone marrow was hypocellular, with a myeloid-to-erythroid ratio of 4–5:1, and megakaryocytes were present at only 1–2 per high-power field. No hemophagocytosis was identified in this bone marrow specimen; however, small foci of necrosis and possible granulomas were observed. The only special stain performed was Foot’s staining, which showed focally mild reticulin increase. The presence of “possible granulomas” in the biopsy report drew our attention and prompted us to reconsider the possibility of Mycobacterium infection and bone marrow infiltration. As we could not exclude the possibility of bone marrow tuberculosis, we conducted another bone marrow aspiration on day 10. The specimens were sent for metagenomic next-generation sequencing (mNGS), TB-DNA and pathological examination. We therefore specifically requested an acid-fast stain and a thorough examination for hemophagocytosis on the bone marrow specimen.

**Figure 3:**
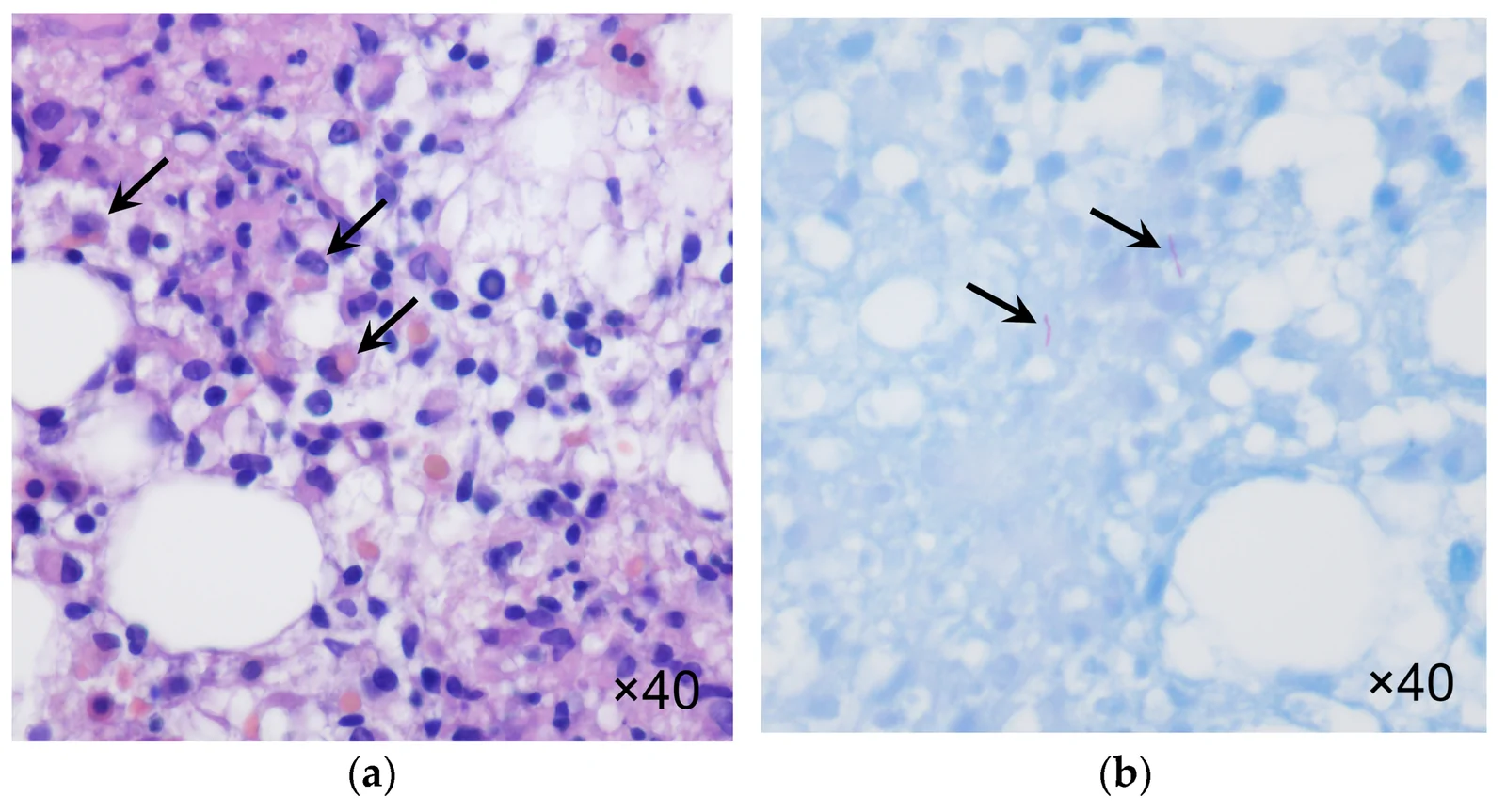
Second bone marrow biopsy of the patient. (**a**) Hematoxylin and eosin (H&E) staining, original magnification ×40. The arrow indicates hemophagocytosis. (**b**) Acid-fast staining, original magnification ×40. The arrows indicate acid-fast bacilli (AFB).

On day 14, the mNGS (Chengdu Huachuang Qide Medical Laboratory, Chengdu, China) report yielded 28 reads of *Mycobacterium tuberculosis*. On day 16, hemophagocytosis was observed on bone marrow smear, and acid-fast bacilli were also identified in bone marrow biopsy (Figure 3). Based on these reports, the patient had met five of the eight criteria of the HLH-2004 guidelines, along with definitive microbiological evidence of tuberculosis. Anti-tuberculosis therapy (ATT) was initiated on day 17 of hospitalization, with isoniazid (300 mg/day), rifampin (600 mg/day), ethambutol (1200 mg/day), and pyrazinamide (1500 mg/day). Dexamethasone was continued as immunomodulatory therapy. By this time, the patient remained in critical condition with profound pancytopenia and transfusion dependence. Despite active treatment, the patient remained severely thrombocytopenic, and he unfortunately died on day 7 of ATT, with intracranial hemorrhage being the presumed cause of death.

## 3. Literature Review

We conducted a literature review of reported TB-HLH cases by searching PubMed using the MESH terms “tuberculosis” and “hemophagocytic lymphohistiocytosis.” Case series without detailed individual patient clinical data, as well as reports for which the full text was unavailable, were excluded. Statistical analyses were performed using SPSS version 27.0(IBM, Armonk, NY, USA). Pearson chi-square tests were used for categorical variables, and a two-sided *p* value < 0.05 was considered statistically significant. A total of 66 cases from 51 reports were included in our review (Table 2). The median age of patients was 38.5 (IQR, 23.75–60.0); 65.2% (43/66) were male.

Disseminated tuberculosis was the most prevalent type of infection, accounting for 78.8% (52/66) of cases; 80.3% (53/66) of cases confirmed the existence of *M. tuberculosis* through culture, NGS, Xpert or PCR, while the diagnosis of remaining cases (13/66) was based on clinical diagnosis.

The overall survival was 57.6% (38/66) in all cases and 52.8% (28/53) in microbiologically confirmed cases. With regard to immunological status, four cases did not document immunological status; 67.7% (42/62) of patients were immunocompetent, and 32.3% (20/62) had various underlying immunocompromising conditions. Among the 53 patients with microbiologically confirmed tuberculosis, immunological status was documented in 50 cases, of which 18 (36.0%) had an underlying immunocompromising condition.

We applied a special notation to findings of bone marrow aspirates and sensitivity of immunological tests (including T-SPOT, TB-IGRA, PPD test and TB-antibody). In 49 cases that documented the outcomes of bone marrow aspirates, 36.7% (18/49) yielded positive results for *M. tuberculosis* by acid-fast smear, culture, Xpert or PCR. Granulomas were identified in 24 bone marrow specimens, and in 21 of these cases—despite negative MTB tests—the histological findings helped maintain the clinical suspicion of MTB infection. Immunological tests were documented in 38 cases, of which 81.6% (31/38) were negative. When the analysis was restricted to cases with microbiologically confirmed MTB infection and available immunological data, negative immunological test results were reported in 90% (27/30) of cases.

Immunomodulatory therapy was administered in 83.3% (55/66) of all cases and 83.0% (44/53) of microbiologically confirmed cases. Of the remaining 11 cases that did not receive immunoregulatory therapy, the reasons were as follows: four critically ill patients refused further treatment, four patients recovered with ATT alone, and three patients died before therapy could be initiated. Timing of ATT was crucial for improving outcomes. In our review, timely ATT was defined as initiation within 5 days of HLH diagnosis. Timely ATT was given in 46 cases, with a survival rate of 78.3% (36/46), while in the other 20 cases in whom ATT was delayed or not given, the survival rate was 15.0% (3/20), significantly lower than that in the timely group (χ^2^ = 21.96, *p* < 0.001). When clinically diagnosed cases were excluded, the conclusion remained unchanged: timely ATT was still associated with better survival (73.5% vs. 15.0%, χ^2^ = 17.86, *p* < 0.001).

## 4. Discussion

We described a rare case of HLH secondary to bone marrow tuberculosis, in which tuberculosis presented in an atypical and misleading manner. This was a diagnostically challenging case: although the negative results of tuberculosis-specific immunological tests argued against tuberculosis, subtle clues—such as the presence of granulomas on bone marrow biopsy—ultimately pointed toward the diagnosis. Had the diagnosis of HLH and bone marrow tuberculosis been established earlier, the patient might have achieved a more favorable outcome.

HLH is a life-threatening hyperinflammatory syndrome. The clinical features of HLH are variable and non-specific, including persistent fever, cytopenia, hepatosplenomegaly, and markedly elevated inflammatory markers such as ferritin and sIL-2R. Its etiology can be broadly classified as primary (genetic) or secondary (acquired), with the latter being far more common in adults and most often triggered by an underlying condition.

Among the environmental triggers of secondary HLH, infection accounts for 24.3% to 53.5% of HLH cases, according to two studies based on large populations [54,55,56,57]. Within the spectrum of infection-induced HLH, Epstein–Barr virus (EBV) is the most notorious, accounting for 22.6% to 82.3% of infection-related HLH [54,56,57,58]. Notably, these proportions vary considerably across studies, largely reflecting differences in age distribution and geographic regions. In contrast, HLH secondary to bacterial infections is less common, with TB being responsible for fewer than 10% of cases in infection-induced HLH [54,56,57].

As a rare trigger of HLH, tuberculosis is easily overlooked and deserves particular attention. TB patients with impaired immune function and disseminated disease are at greater risk of developing secondary HLH [59]. This is consistent with our literature review, which indicates that tuberculosis causing secondary HLH is typically disseminated. Bone marrow tuberculosis represents a subtype of disseminated disease; though extremely rare, isolated bone marrow involvement without other organ infiltration is also regarded as disseminated tuberculosis in the literature. Typical imaging findings such as miliary nodules make the diagnosis of disseminated pulmonary tuberculosis relatively straightforward. In contrast, isolated bone marrow tuberculosis and cases without clear clues of TB are far more elusive and may escape detection by routine peripheral blood examinations. The clinical features of bone marrow tuberculosis largely overlap with those of HLH, including fever, cytopenia, and hepatosplenomegaly, making early recognition particularly challenging [60].

This diagnostic difficulty is compounded by the unreliability of conventional immunological tests in the setting of HLH. Immunological examinations, such as TB-IGRA, T-SPOT and TST, may facilitate early diagnosis of TB. However, in many of the TB-HLH cases we reviewed, those immune-based assays were mostly negative. Immunological tests for tuberculosis are based on the detection of host immune responses. TST measures delayed-type hypersensitivity to PPD, while IGRAs quantify IFN-γ release from T cells stimulated with MTB-specific antigens [61,62]. Both tests therefore require intact cellular immunity. It has also been reported that T-SPOT has a decreased accuracy in extrapulmonary TB [63]. In patients with immune suppression, including HLH-related immune dysfunction, these tests may yield false negative results [61,62]; our literature review also showed that negative immunological test results were frequently reported among microbiologically confirmed cases with available data (27 of 30). For those patients, molecular examinations, including Xpert MTB/RIF or NGS, are better choices, with increased sensitivity and potential of early diagnosis [64,65]. We examined the association between WBC level and immunological test positivity. Lymphocyte counts were not consistently reported across cases, so WBC was used as a surrogate marker of immune status. However, no statistically significant association was found. Negative immunological results were the main reason why most cases did not receive timely ATT. In some cases [3,52], tuberculosis was not even considered until mycobacterial culture yielded *M. tuberculosis*, resulting in further treatment delay.

In many cases, the diagnosis of HLH precedes that of TB, and unreliable immunological tests often misdirect the diagnostic workup. Huang et al. reported a similar case of a patient with a primary immunological defect, in whom initial serum investigations were all negative. *Mycobacterium tuberculosis* was identified by culture three weeks after the initiation of anti-HLH therapy, resulting in a significant delay in anti-tuberculosis treatment [66]. In contrast, Zhu et al. reported a case of TB-HLH in which T-SPOT was initially negative in the setting of neutropenia, but converted to positive after neutrophil recovery, prompting early initiation of anti-tuberculosis therapy and immunomodulation [6]. Conversely, in the case reported by Trovik et al., an initial positive TB-IGRA turned negative as the patient developed progressive neutropenia during the course of HLH [23]. Both cases reported a favorable prognosis. A TB-HLH case in NEJM’s Images in Clinical Medicine also had an indeterminate TB-IGRA and no initial TB evidence; both TB and HLH were eventually confirmed by bone marrow biopsy [25]. Taken together, these observations indicate that immunological tests for tuberculosis are unreliable in the setting of HLH and should not be used to exclude the diagnosis, which is an important lesson reinforced by our case.

The principles of HLH management emphasize prompt control of the life-threatening hyperinflammatory state. Among these, the identification and treatment of the underlying trigger is the cornerstone of durable disease control and prevention of relapse. After initial control of the hyperinflammatory state, targeting the underlying trigger may be more important than HLH-specific therapy. Our review showed that several cases with controlled tuberculosis had favorable outcomes even without immunoregulatory therapy [27,35,41,47], and that prognosis was closely linked to the timing of ATT. In patients with suspected TB-HLH, early diagnosis and timely initiation of ATT are critical for improving outcomes [41,52]. Both ATT and IVIG were associated with reduced mortality, and the addition of HLH-directed therapy further improved outcomes [41,60]. The findings of our literature review are also in line with that conclusion: timely ATT was significantly associated with lower mortality. However, most included cases did not report detailed timelines, so actual time intervals could not be analyzed. Given the lack of standardized definitions and the limited available literature, we empirically used a 5-day cutoff, which allowed us to classify treatment timing in most cases. We must emphasize that this 5-day cutoff is empirical and may introduce misclassification bias; a standardized definition of timely ATT in TB-HLH requires further prospective studies.

Accurate and early diagnosis is the prerequisite for timely ATT. However, as discussed above, identifying tuberculosis early as the trigger in TB-HLH patients without typical findings is extremely difficult. The need for early diagnosis highlights the importance of performing bone marrow aspiration in suspected TB-HLH cases. Rapid molecular testing on bone marrow aspirate or biopsy, such as Xpert MTB/RIF and metagenomic next-generation sequencing (mNGS), can substantially shorten the time to diagnosis compared with conventional culture, which may take several weeks. In patients with suspected TB-HLH, where diagnostic delay is associated with poor outcomes, these assays should be considered early in the diagnostic workup. Bone marrow biopsy is also beneficial in establishing the diagnosis of HLH. Evidence of hemophagocytosis is crucial for HLH diagnosis, and direct detection of pathogens by staining helps establish the etiological diagnosis of HLH. HLH can also be triggered by bone marrow infiltration from other bacterial pathogens, and staining of bone marrow specimens may also help identify the underlying etiology in such cases [67]. Even when pathogens are not directly identified in the biopsy specimen, features such as granulomatous changes may also prompt further investigation for specific pathogens and subsequent empirical therapy. As our literature review revealed, though acid-fast bacilli were absent, the presence of granulomas served as a clue to mycobacterial infection in 21 specimens. Bone marrow biopsy is considered to have high sensitivity for detecting hemophagocytosis in TB-HLH cases [41]. However, hemophagocytosis was not identified in the initial bone marrow specimen in our case, which left us uncertain about the diagnosis of HLH. However, based on persistent fever, cytopenia, markedly elevated ferritin and sIL-2R levels, and especially granulomas in the biopsy, we maintained a high clinical suspicion. A repeat bone marrow examination was performed, and hemophagocytosis was eventually observed—but only after we specifically alerted the pathologist to look for it. This case illustrates that the absence of hemophagocytosis on an initial bone marrow examination does not rule out HLH when other clinical and laboratory criteria are strongly suggestive. It also highlights the importance of close communication between clinicians and pathologists, as subtle hemophagocytic features may otherwise be overlooked.

Earlier diagnosis would have enabled earlier ATT, which might have improved the prognosis. Whether earlier diagnosis and initiation of ATT would have altered the outcome remains uncertain in this single fatal case. The patient’s initial presentation was already complicated by severe pancytopenia and critical illness, both of which likely contributed to the fatal intracranial hemorrhage. While earlier diagnosis may have enabled earlier ATT and potentially mitigated disease progression, this remains a speculative hypothesis rather than a conclusion supported by the present case. We hope that this instructive case, together with our literature review, will provide clinicians with new insights into TB-HLH and optimize the diagnostic approach to identifying the underlying trigger of HLH.

Our report has several limitations. Our literature review is largely based on published case reports and case series, and patients with unusual presentations, diagnostic challenges, or adverse outcomes are more likely to be reported. This selection and reporting bias may have influenced our estimates of mortality, diagnostic test performance, and the association between treatment timing and outcomes. Future prospective cohort studies are needed to provide more reliable estimates in patients with TB-HLH.

## 5. Conclusions

TB is an uncommon trigger of HLH, with insidious onset and prolonged treatment course. Its clinical manifestations may overlap with those of HLH, leading to delayed diagnosis and treatment, and consequently an unfavorable prognosis. Immunological tests for tuberculosis are unreliable in the context of HLH; early application of molecular diagnostic tools—such as PCR, NGS, and Xpert—is helpful for early diagnosis. Bone marrow biopsy should be considered in patients with unexplained HLH, particularly when conventional diagnostic workup is unrevealing, as it serves a dual purpose: confirming HLH and identifying its underlying etiology. When granulomatous changes are identified in a clinical context suggestive of tuberculosis, empirical anti-tuberculosis therapy may be considered, although other causes of granulomatous inflammation should be excluded. In addition, close communication between clinicians and pathologists is critical, as hemophagocytosis may be subtle and easily overlooked on initial examination.

## Figures and Tables

**Table 1:** HLH-2004 diagnostic criteria fulfilled by the patient.

HLH-2004 Criteria	Met Upon First BMS (4/8)	Met Upon Second BMS (5/8)
Fever (≥38.5 °C, ≥7 days)	√	√
Splenomegaly	×	×
Cytopenia (≥2 lineages)	√	√
Hypertriglyceridemia or hypofibrinogenemia	×	×
Hemophagocytosis	×	√
Low/absent NK cell activity	× (not tested)	× (not tested)
Ferritin ≥500 μg/L	√	√
Elevated sIL-2R (>2400 U/mL)	√	√

**Table 2:** Literature review of TB-HLH cases (including ours).

Author	Year	Age	Gender	Immune Background	Tuberculosis	Clinical Diagnosed	Pathogens Detected in	Bone Marrow Acid-Fast Smear	Granuloma in Biopsy	Immunological Tests ^1^	WBC (×10^9^/L)	ATT ^2^	Outcome
our case		34	Male	immunocompetent	bone marrow	no	Bone marrow	+ ^3^	+	− ^3^	0.37	delayed	died
Pal [2]	2026	68	male	NM	pleurisy	no	pleural fluid	NM	NM	NM	NM	timely	alive
		36	male	NM	pleurisy	no	pleural fluid	NM	NM	NM	NM	timely	alive
		35	male	NM	pleurisy	yes	none	NM	NM	NM	3.1	timely	alive
		23	female	NM	disseminated	no	sputum	NM	NM	NM	8.8	timely	died
Nakahara [3]	2026	70	female	immunosuppression	disseminated	no	blood, abscess, BALF	−	−	+	2.7	delayed	died
Jin [4]	2025	52	male	immunocompetent	disseminated	no	bone marrow	−	+	−	NM	delayed	alive
Fu [5]	2025	62	male	AIDS	lymphadenitis	no	lymph node	−	−	−	2.6	timely	alive
Zhu [6]	2025	60	male	immunocompetent	disseminated	no	CT-guided T9 biopsy	−	−	− then +	1.75	timely	alive
Assaf [7]	2025	57	female	immunocompetent	disseminated	no	BALF	NM	NM	−	1.16	delayed	died
Seers [8]	2025	63	male	immunosuppression	disseminated	no	BALF, bone marrow	+	+	NM	0.8	timely	alive
Wu [9]	2024	28	male	GATA2 mutation	disseminated	no	lymph node, blood	−	−	−	1.5	timely	alive
Nielsen [10]	2024	50	female	immunosuppression	disseminated	no	lymph node, bone marrow, sputum	+	−	−	NM	timely	alive
Liu [11]	2024	87	male	immunocompetent	disseminated	no	sputum, abscess	−	+	+	2.26	delayed	alive
Vidhale [12]	2023	20	male	immunocompetent	pulmonary	yes	none	NM	NM	+	9.55	timely	alive
Nazhand [13]	2023	60	male	immunosuppression	disseminated	no	BALF, bone marrow	+	+	NM	NM	timely	died
Ing [14]	2023	53	male	immunocompetent	disseminated	no	BALF, bone marrow	+	−	NM	3.7	timely	alive
Uryu [15]	2023	76	female	immunocompetent	disseminated	no	sputum, blood, bone marrow	+	+	−	5.8	timely	alive
Ferreira [16]	2022	52	male	immunosuppression	disseminated	no	CSF	−	−	NM	0.93	delayed	died
Cerme [17]	2022	46	male	familial mediterranean fever	disseminated	no	blood, BALF	+	+	−	2.7	delayed	died
Wang [18,19]	2022	28	male	immunocompetent	disseminated	no	blood, BALF	−	−	−	13.8	timely	alive
Li [20]	2022	28	male	immunocompetent	disseminated	yes	none	NM	NM	+	5.89	timely	alive
Costa [21]	2021	76	male	immunosuppression	disseminated	no	lymph node	−	−	NM	NM	timely	alive
Shi [22]	2021	26	female	immunosuppression	disseminated	no	pleural fluid, sputum, BALF, blood	−	−	±	13.9	timely	alive
Trovik [23]	2020	54	male	immunocompetent	disseminated	no	BALF, bone marrow	+	+	+ then −	10.4	timely	alive
Wang [24]	2020	36	male	immunocompetent	pulmonary	yes	lung biopsy	−	−	+	5.32	timely	alive
Sacks [25]	2020	63	male	immunocompetent	disseminated	no	sputum, bone marrow	+	+	±	NM	timely	died
Ranjan [26]	2020	40	male	immunocompetent	disseminated	yes	none	NM	NM	−	3.2	timely	alive
Geerdes-Fenge [27]	2019	22	male	immunocompetent	disseminated	no	peritoneal biopsy	NM	NM	−	NM	timely	alive
Mbizvo [28]	2019	54	female	immunocompetent	disseminated	no	blood, sputum, CSF, bone marrow	+	+	NM	0.1	timely	alive
Asaji [29]	2017	19	female	immunocompetent	disseminated	no	BALF, sputum, urine	−	+	±	7.54	timely	alive
Fernández [30]	2017	20	female	immunocompetent	disseminated	no	bone marrow, placenta	+	+	NM	4.6	timely	alive
Long [31]	2017	35	female	immunosuppression	disseminated	no	blood	−	−	NM	16.7	timely	alive
Haque [32]	2017	23	male	immunocompetent	disseminated	yes	none	−	−	+	NM	timely	alive
Chen [33]	2017	20	male	immunocompetent	bone marrow	yes	none	−	+	−	3.8	timely	alive
		57	male	immunocompetent	disseminated	no	BALF	−	+	−	NM	timely	died
Zhang [34]	2017	60	female	immunocompetent	pulmonary	no	NM	NM	NM	−	NM	delayed	died
		31	female	immunocompetent	meningitis	no	NM	NM	NM	−	NM	delayed	died
		78	female	immunocompetent	pulmonary	no	NM	NM	NM	−	NM	delayed	died
		32	male	immunocompetent	disseminated	no	NM	NM	NM	−	NM	timely	alive
		37	male	immunocompetent	disseminated	no	NM	NM	NM	−	NM	delayed	died
		28	female	immunocompetent	pulmonary	no	NM	NM	NM	−	NM	timely	alive
		23	female	immunocompetent	peritonitis	no	NM	NM	NM	−	NM	timely	died
		60	female	immunocompetent	tuberculosis	no	NM	NM	NM	−	NM	delayed	died
Sass [35]	2016	1	female	immunocompetent	disseminated	no	cardiac vegetation, gastric fluid, bone marrow	+	NM	NM	14.2	timely	alive
Osowicki [36]	2016	6w	male	infant	disseminated	no	BALF	−	NM	−	1.7	timely	alive
Tesi [37]	2015	4	female	IFN-γ receptor 1 deficiency	disseminated	no	blood, CSF	−	−	−	NM	timely	died
Rathnayake [38]	2015	40	female	immunocompetent	disseminated	yes	none	−	−	−	1.7	timely	alive
Koulmane Laxminarayana [39]	2015	56	male	CKD5	disseminated	yes	none	−	+	NM	NM	delayed	died
		50	male	CKD5	disseminated	no	bone marrow	+	+	NM	NM	delayed	died
		36	male	CKD5	disseminated	no	bone marrow	+	+	NM	NM	delayed	died
Hui [40]	2015	42	male	immunocompetent	disseminated	no	BALF	−	+	+	15.2	delayed	alive
Padhi [41]	2015	17	male	immunocompetent	disseminated	yes	none	−	+	NM	1.3	timely	alive
Dey [42]	2014	2m	male	infant	disseminated	yes	BALF	−	−	NM	1.58	timely	died
Cherif [43]	2013	18	female	immunocompetent	disseminated	yes	none	−	−	−	2.32	timely	alive
Medina [44]	2013	63	male	immunosuppression	disseminated	no	bone marrow, blood	+	−	−	1	delayed	died
Naha [45]	2013	27	male	immunocompetent	disseminated	no	lymph node aspirate, bone marrow	−	+	NM	0.8	timely	died
Li [46]	2012	16	male	immunocompetent	disseminated	yes	sputum, peritoneal fluid	−	+	NM	3	timely	alive
Shea [47]	2012	78	female	immunocompetent	disseminated	no	blood, sputum, urine, BALF	−	−	NM	1.7	timely	alive
		80	male	immunocompetent	disseminated	no	gastric fluid, bone marrow, urine	+	+	NM	1.77	timely	died
Aggarwal [48]	2012	34	male	immunocompetent	disseminated	no	pleural fluid	−	−	NM	1.2	delayed	died
Maheshwari [49]	2012	3w	female	infant	tuberculosis	no	BALF	−	−	NM	NM	timely	alive
Lee [50]	2008	63	male	immunocompetent	disseminated	no	bone marrow, sputum	+	+	NM	9.2	timely	died
Balasubramanian [51]	2008	52d	male	infant	disseminated	no	gastric fluid	−	−	NM	59.5	delayed	died
Brastianos [52]	2006	28	male	immunocompetent	disseminated	no	liver	−	−	−	1.34	delayed	died
Claessens [53]	2006	68	male	immunocompetent	disseminated	no	bone marrow	+	+	NM	3.5	timely	alive
		67	female	immunocompetent	disseminated	no	bone marrow, sputum, BALF	+	+	NM	18.7	delayed	died

ATT, anti-tuberculosis therapy; BALF, bronchoalveolar lavage fluid; CKD5, chronic kidney disease stage 5; NM, not mentioned; WBC, white blood cell count. ^1^ Including PPD test, TB-IGRA, T-SPOT, and TB antibody assays. ^2^ Treatment delay was defined as failure to initiate ATT within 5 days of meeting HLH diagnostic criteria. ^3^ In this table, “+” and “−” indicate positive and negative test results, respectively.

## Data Availability

All relevant data supporting the findings of this study are included within the article. No additional data were generated or analyzed for this case report and literature review. The datasets used are not publicly available due to patient confidentiality and ethical restrictions.

## Abbreviations

The following abbreviations are used in this manuscript:

AFB	acid-fast bacilli
ALT	alanine aminotransferase
AST	aspartate aminotransferase
ATT	anti-tuberculosis therapy
BMS	bone marrow aspiration
CT	computed tomography
HLH	hemophagocytic lymphohistiocytosis
IFN-γ	interferon-gamma
IGRA	interferon-gamma release assay
sIL-2R	soluble interleukin-2 receptor
IQR	interquartile range
IVIG	intravenous immunoglobulin
LDH	lactate dehydrogenase
mNGS	metagenomic next-generation sequencing
MTB	*Mycobacterium tuberculosis*
NGS	next-generation sequencing
NK	natural killer
PCR	polymerase chain reaction
PPD	purified protein derivative
TB	tuberculosis
TB-HLH	tuberculosis-induced hemophagocytic lymphohistiocytosis
TST	tuberculin skin test
WBC	white blood cell
